# Multi-parametric MRI-based radiomics nomogram for predicting lymphovascular space invasion in early-stage cervical adenocarcinoma

**DOI:** 10.3389/fonc.2025.1612691

**Published:** 2025-08-21

**Authors:** Ke-Ying Wang, Mei-Ling Xiao, Yu-Han Fang, Jie-Jun Cheng, Zi-Jing Lin, Ying Li, Jin-Wei Qiang

**Affiliations:** ^1^ Department of Radiology, Jinshan Hospital, Fudan University, Shanghai, China; ^2^ Department of Nuclear Medicine and PET Center, The Second Affiliated Hospital of Zhejiang University School of Medicine, Hangzhou, China; ^3^ Department of Radiology, Shanghai First Maternity and Infant Hospital, Tongji University School of Medicine, Shanghai, China

**Keywords:** cervical adenocarcinoma, lymphovascular space invasion, magnetic resonance imaging, radiomics, nomogram

## Abstract

**Purpose:**

To develop a magnetic resonance imaging (MRI)-based radiomics nomogram to predict lymphovascular space invasion (LVSI) status in patients with early-stage cervical adenocarcinoma (CAC).

**Methods:**

Clinicopathological and MRI data from 310 patients with histopathologically confirmed early-stage CAC were retrospectively analyzed. Patients were divided into training (n = 186) and validation (n = 124) cohorts. Tumor volumes of interest (VOIs) were segmented on T2-weighted imaging (FS-T2WI) and aligned to diffusion-weighted imaging (DWI), apparent diffusion coefficient (ADC) maps, and T1-weighted imaging (CE-T1WI) sequences. Radiomics features were extracted and screened using Pearson correlation and least absolute shrinkage and selection operator (LASSO) regression, and a radscore was calculated for each patient. Multivariate logistic regression identified clinical risk factors, and a radiomics nomogram was constructed by integrating the radscore with clinical risk factors. Receiver operating characteristic (ROC) curves and areas under the curve (AUCs) were used to evaluate the performance of the clinical model, radiomics model, and nomogram. Decision curve analysis was used to assesses the clinical utility of the nomogram.

**Results:**

Seventeen radiomics features were selected to construct the radscore. Menopause and tumor diameter were identified as independent clinical risk factors for LVSI. The radiomics nomogram achieved AUCs of 0.80 (95% CI: 0.74-0.86) and 0.78 (95% CI: 0.69-0.86) in the training and validation cohorts, outperforming the clinical model (AUCs: 0.69 and 0.62) and comparable to the radiomics model (AUCs: 0.79 and 0.78). Decision curve analysis showed the nomogram provided clinical benefit.

**Conclusions:**

The radiomics nomogram, integrating radiomic features and clinical risk factors, could be used to predict LVSI status in early-stage CAC accurately, supporting preoperative clinical decision-making.

## Introduction

Cervical cancer (CC) is the fourth most common cancer and cause of death among women, with approximately 570,000 new cases and 310,000 deaths annually worldwide (1). The 5-year overall survival rate of patients with early CC after surgery is greater than 65%, but the recurrence rate is as high as 30% (2, 3). Cervical adenocarcinoma (CAC) is the second most prevalent histological subtype of CC following cervical squamous cell carcinoma (SCC, the most prevalent histological subtype of CC) (4). In recent years, with the popularization of CC screening and the application of the HPV vaccine, the mortality rate of SCC has been decreased (5, 6). However, the incidence and mortality of CAC are increasing annually (7).

The prognosis and selection of postoperative treatment plans for patients with early CC following radical resection are primarily contingent upon postoperative pathological risk factors influencing recurrence. Risk factors such as the histological type of the tumor, tumor size, positive surgical margin, involvement of the lower uterine segment, lymphovascular space invasion (LVSI), depth of stromal invasion, parametrial invasion, and lymph node metastasis (LNM) are associated with a higher recurrence rate and poorer survival outcomes in patients (8–10). Most patients with stage IB-IIA cervical cancer, based on the International Federation of Gynecology and Obstetrics (FIGO) staging system, are treated with hysterectomy-based surgery with pelvic lymph node dissection (PLND). Adjuvant radiotherapy (RT) or concurrent chemoradiotherapy (CCRT) after operation can improve progression-free survival and overall survival for patients with early stage CC with these risk factors (11). Therefore, a preoperative and noninvasive assessment to predict adverse pathologic factors is of great importance to optimize a treatment plan to lower the incidence of post-treatment morbidity and improve the quality of life.

Lymphovascular space invasion (LVSI) is defined as the presence of malignant cells within endothelial-lined vascular or lymphatic spaces outside of the primary invasive tumor, which plays a crucial role in tumor invasion and metastasis (12). Previous study showed that, LVSI is an essential prognostic factor for recurrence as well as overall survival in patients with early-stage CC (13). For CC patients with early-stage without LVSI, conization is recommended. However, for CC patients with early-stage with LVSI, radical hysterectomy and pelvic lymph node dissection is recommended (14). Furthermore, due to the more aggressive characteristics of CAC, it is important to predict LVSI in CAC preoperative. However, at present, most reported studies of LVSI in CC are based on SCC. Therefore, it is of great clinical usefulness to establish a stable and reliable prediction model for LVSI in early-stage CAC.

Magnetic resonance imaging (MRI) is a noninvasive imaging modality widely used in the assessment of female pelvic tumors (15, 16). However, for conventional MRI, the assessment of the imaging results performed by radiologists. LVSI status is unable to be assessed. Radiomics is an innovative technology, which transform the visual image information into high-dimensional (17). Radiomics has been gradually applied to predict LVSI status in gynecological pelvic tumors (18–20). While radiomics-based prediction of LVSI has been extensively explored in SCC, limited attention has been given to CAC, a histologically distinct and more aggressive subtype. Given CAC’s glandular architecture and higher heterogeneity, radiomic patterns may differ significantly.

We assumed that radiomics could be useful in predicting LVSI in CAC preoperatively. Thus, this study aimed to establish and validate a multi-parametric MRI-based nomogram for preoperatively predicting LVSI status in patients with early-stage CAC.

## Materials and methods

### Ethnic consideration

This study was reviewed and approved by the Institutional Review Board of Jinshan Hospital, Fudan University (No. JIEC2024-S45). Informed consent was waived for all patients due to the retrospective nature of the study. The methods conducted adhered to relevant guidelines and regulations.

### Patients

A total of 375 patients with CAC confirmed by histology from June 2018 to September 2021 were screened in the medical imaging information system. The clinicopathological and MRI data of patients were analyzed.

The inclusion criteria for early-stage CAC were as follows: Histopathologically confirmed early-stage CAC (FIGO stage IB-IIB). Availability of clinicopathological information. Patients underwent surgery within one month following the MRI examination. MRI sequences included fat-saturated T2-weighted imaging (FS-T2WI), T1-weighted imaging (T1WI), diffusion-weighted imaging (DWI), apparent diffusion coefficient (ADC) maps, and contrast-enhanced (CE)-T1WI. Exclusion criteria were as follows: Tumors too small to be seen (lesion diameter < 1 cm). Poor image quality with obvious artifacts. Patients receiving adjuvant radiotherapy or chemotherapy before surgery. Finally, a total of 310 early-stage CAC patients were enrolled in the study. Among them, 186 patients were assigned to the training cohort, while 124 patients were assigned to the validation cohort in a 6:4 ratio randomly.

### Clinical information

The demographic and clinicopathological data of all enrolled patients were reviewed, including age, reproductive history, family history of malignancy, menopausal status, FIGO stage, LVSI status on pathology, tumor diameter, and parametrial invasion status on MRI. Parametrial invasion was assessed based on the disruption of the normal hypointense stromal ring on T2-weighted imaging (T2WI). Loss of the hypointense stromal ring continuity, direct tumor extension into the parametrial fat, or irregularity in the surrounding tissue signal were considered indicative of invasion. The MRI features were interpreted by two radiologists (radiologist 1 and radiologist 2, with 3 and 15 years of experience in gynecological imaging, respectively) to reach consensus, and confirmed by radiologist 3 (with 25 years of experience in gynecological imaging) in cases of disagreement.

### Image scan and segmentation

MRI was performed on a 3.0 T MR system (Verio Siemens Erlangen Germany). MRI sequences and parameters are listed in Appendix I. Using ITK-SNAP software (http://www.itksnap.org), the regions of interest (ROIs) were manually drawn along the tumor margin on each FS-T2WI slice by radiologist 1 and automatically matched to DWI, ADC maps, and delay-phase CE-T1WI sequences. The volume of interest (VOI) of the tumor was automatically displayed following ROI delineation. One month later, 30 patients were randomly selected for ROI delineation by radiologist 1 and radiologist 2. Interclass and intraclass correlation coefficients (ICCs) were calculated to evaluate the reproducibility of radiomics features.

### Image feature extraction and selection

The VOIs were imported into the Pyradiomics (version 3.2.0) toolkit, which runs in a Python (version 3.9.0) environment, to extract radiomics features. Before feature extraction, all images were resampled to an isotropic voxel size of 1×1×1 mm^³^ and underwent Z-score normalization to standardize intensity distributions across sequences. These preprocessing steps were performed to minimize scanner-related variability and systematic bias. Features with high collinearity (Pearson r > 0.9) were filtered prior to LASSO regression. Features with an ICC > 0.75 were considered to have satisfactory reproducibility and were retained. Pearson’s correlation was then utilized to detect redundant features, and if the correlation coefficient was > 0.9 for both features, the one with the larger mean absolute coefficient was eliminated. The least absolute shrinkage and selection operator (LASSO) regression was performed to select radiomics features associated with LVSI in early-stage CAC. Penalty parameter adjustment was performed using 10-fold cross-validation to select nonzero coefficient features associated with LVSI.

### Nomogram development and evaluation

Univariate and multivariate logistic regression analyses were performed, with a stepwise backward selection method was applied to screen clinical independent risk factors for CAC LVSI. The stopping criterion was Akaike’s likelihood ratio test. The final inclusion of clinical risk factors was based on relevant studies of CAC LVSI risk factors. The radscore was computed for each patient using the selected radiomics features and their corresponding regression coefficients. A radiomics nomogram was established by combining the radscore with selected clinical risk factors. Receiver operating characteristic (ROC) curves with the area under the curves (AUC) were employed to assess the diagnostic efficacy of the models. Calibration curves were used to evaluate the goodness of fit of the nomogram (agreement between predicted and observed results), and the clinical utility of the nomogram was assessed using decision curve analysis.

### Statistical analysis

Statistical analyses were performed using R software (version 4.4.0; http://www.R-project.org). Quantitative variables were presented as mean ± standard deviation (SD) for normal distributions or as median and interquartile range for non-normal distributions, and were compared using the Student’s t-test or Mann-Whitney U test. Qualitative variables were compared using the Chi-square test or Fisher’s exact test. DeLong’s test was used to compare the diagnostic performance between the radiomics nomogram and radiologists. A p-value ≤ 0.05 was considered statistically significant.

## Results

### Clinical characteristics

**Figure 1:**
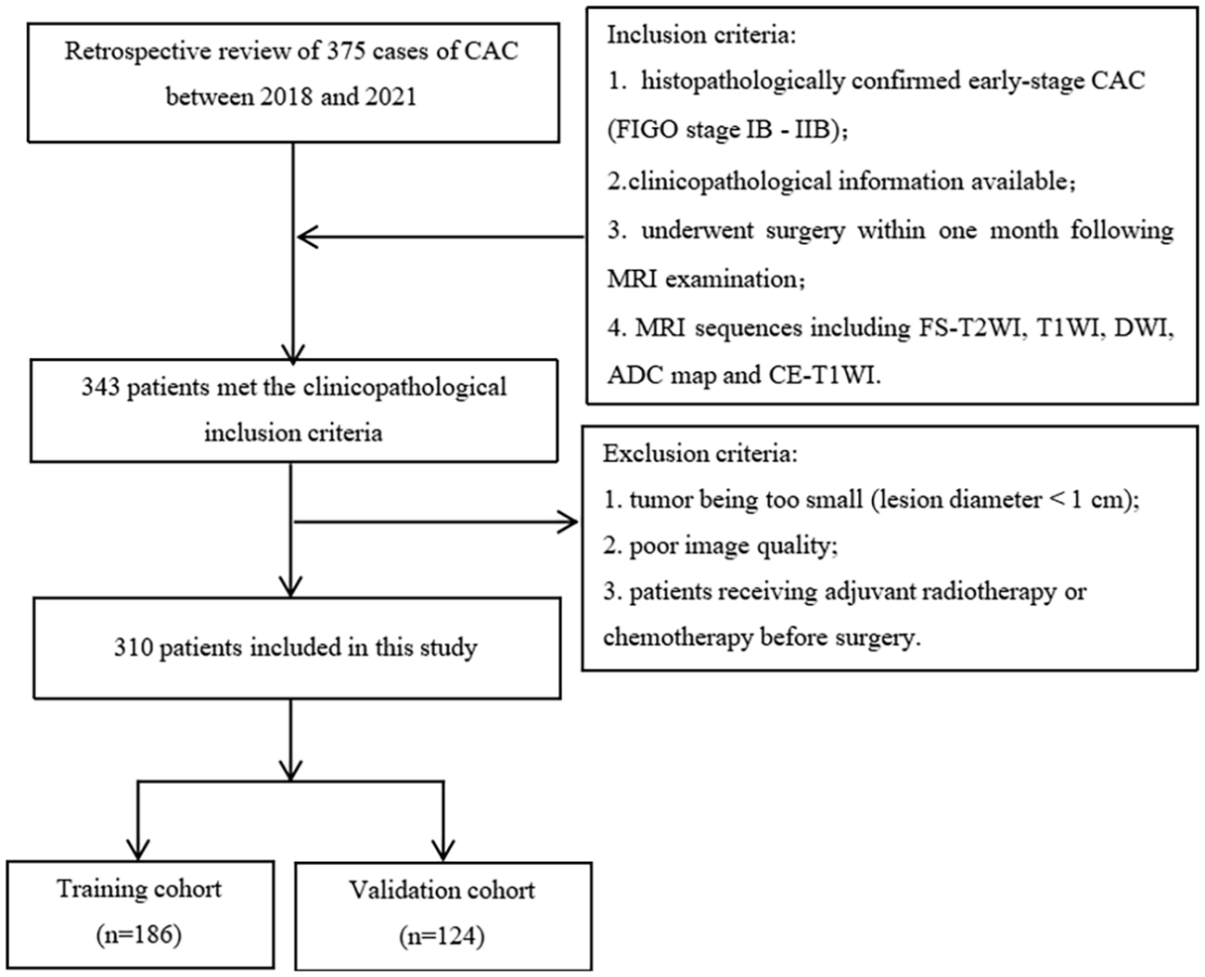
Flowchart of patient inclusion and exclusion and grouping. (CAC, cervical adenocarcinoma; MRI, magnetic resonance imaging; FS-T2WI, fat-suppressed T2-weighted imaging; T1WI, T1-weighted imaging; DWI, diffusion-weighted imaging; ADC, apparent diffusion coefficient; CE-T1WI, contrast-enhanced T1WI).

Of the 310 patients with early-stage CAC, there were 186 LVSI (+) and 124 LVSI (-) cases confirmed by surgical pathology. The clinicopathological features of patients with LVSI (+) and LVSI (-) are listed in **Table 1**. The flowchart of inclusion, exclusion, and grouping of patients with early-stage CAC is shown in **Figure 1**.

**Table 1:** Comparison of clinicopathologic features between LVSI (+) and LVSI (-) in patients with early-stage CAC.

Characteristics	Training cohort			Validation cohort		
	LVSI(+) (N=110)	LVSI(-) (N=76)	P-value	LVSI(+) (N=110)	LVSI(-) (N=76)	P-value
Age (y)	48 ± 10.9	46 ± 8.6	0.191	48 ± 10.3	48 ± 9.7	0.966
Family CA history	7 (6.4%)	2 (2.6%)	0.413	8 (10.5%)	11 (22.9%)	0.107
Reproductive history	98 (89.1%)	73 (96.1%)	0.150	74 (97.4%)	44 (91.7%)	0.312
Menopause	53 (48.2%)	22 (28.9%)	0.013	31(40.8%)	21 (43.8%)	0.009
FIGO stage I/II	83/27 (75.5%/24.5%)	64/12 (84.2%/15.8%)	0.208	56/20 (73.7%/26.3%)	37/11 (77.1%/22.9%)	0.831
Tumor diameter (cm)	3.9 ± 1.1	3.3 ± 1.2	0.002	3.7 ± 1.2	3.2 ± 1.0	0.017
PMIMR	28 (25.5%)	9 (11.8%)	0.036	11 (14.5%)	4 (8.3%)	0.046
DCSRMR	73 (66.4%)	32 (42.1%)	0.002	43 (56.6%)	26 (54.2%)	0.038

CAC, cervical adenocarcinoma; LVSI, lymph-vascular space invasion; FIGO, International Federation of Gynecology and Obstetrics; PMIMR, parametrial invasion on MRI; DCSRMR, disruption of the cervical stromal ring on MRI.

Univariate analysis was used to screen for potential clinical predictors of LVSI. Variables with P < 0.05 were further assessed using multivariate logistic regression to identify independent risk factors. Univariate analysis revealed that menopause, tumor diameter, parametrial invasion on MRI (PMIMR), and disruption of the cervical stromal ring on MRI (DCSRMR) were four differential features between the LVSI (+) and LVSI (-) groups in the training and validation cohorts (all P < 0.05). Multivariate logistic regression analysis (**Table 2**) demonstrated that menopause and tumor diameter were independent risk factors for LVSI in early-stage CAC. A clinical model was developed based on these two clinical independent risk factors.

**Table 2:** Multivariate logistic regression analysis of risk factors for LVSI in cervical adenocarcinoma in the training group.

Characteristics	Multivariate logistic regression analysis			
	Estimate	Std. error	t value	Pr(>|t|)
Menopause	0.29	0.11	2.62	0.009
Tumor diameter	0.09	0.04	2.50	0.013
PMIMR	-0.01	0.11	-0.11	0.911
DCSRMR	- 0.15	0.08	- 1.90	0.060

### Radiomic feature selection and radscore construction

**Figure 2:**
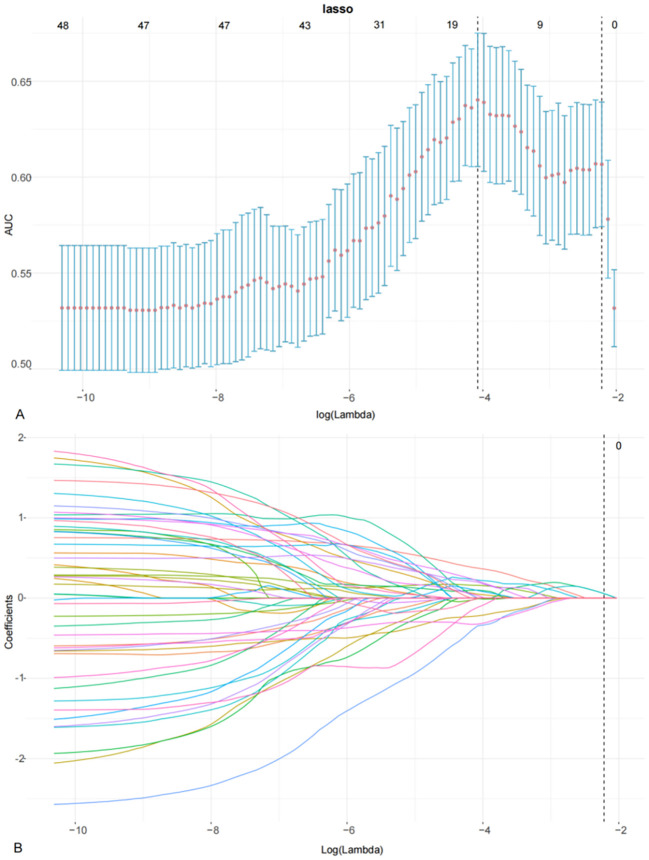
LASSO coefficient profiles using the LASSO algorithm to select 17 radiomics features **(A)** using 10-fold cross-validation to select the parameters λ **(B)**. A vertical line is drawn at the optimal value to generate 17 features with non-zero coefficients.

**Figure 3:**
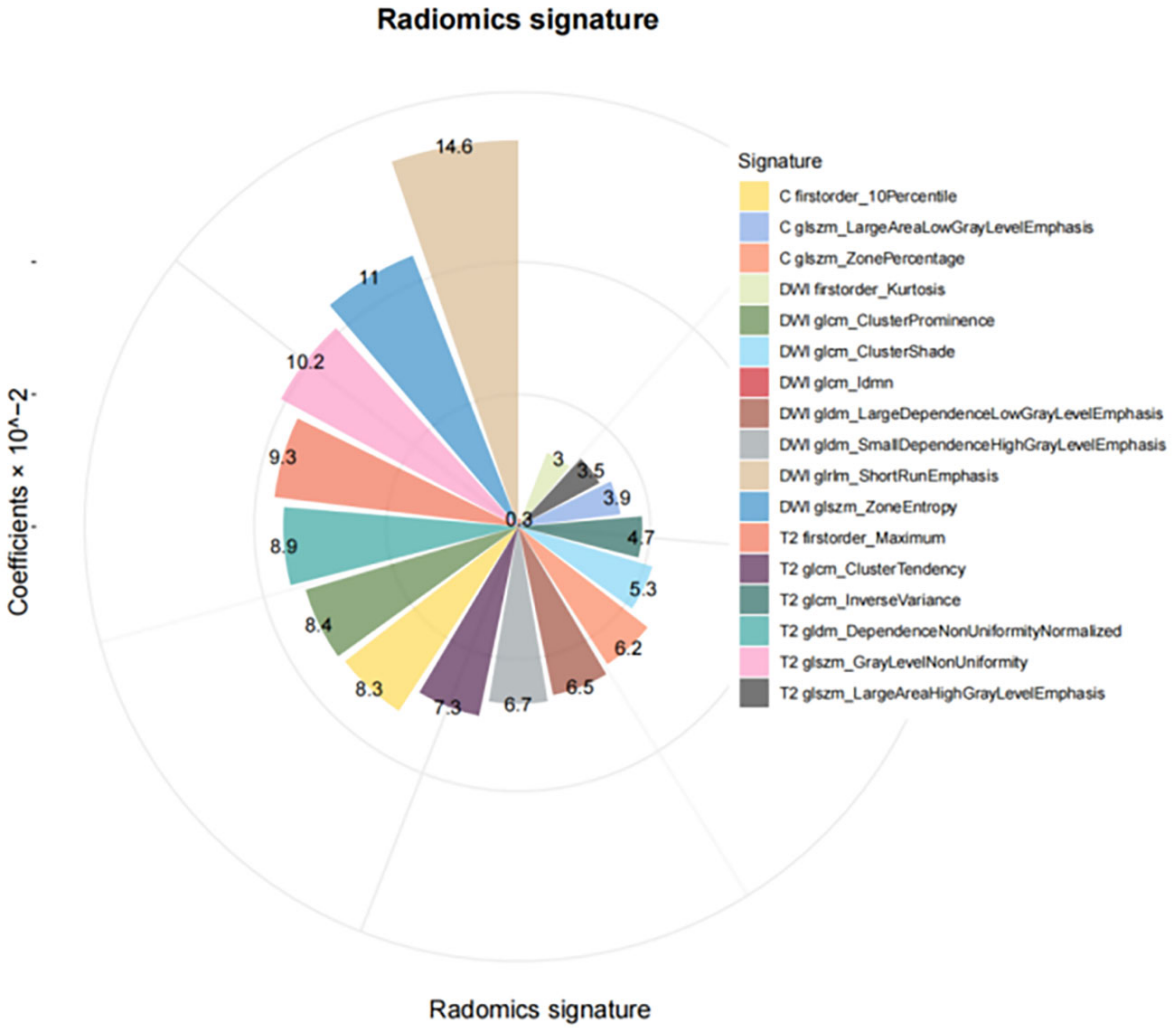
Selected 17 radiomics features and their respective non-zero weighting coefficient.


In the training cohort, 405 features were identified on DWI, FS-T2WI, and delay-phase CE-T1WI sequences. Following feature selection, 17 radiomics features were selected, as illustrated in **Figure 2**, and the diagnostic efficacy of the 17 radiomics features were showed in **Supplementary Table 1**. These features included first-order features based on the original image and texture features, including the gray size region matrix (GLSZM), gray level co-occurrence matrix (GLCM), gray level dependency matrix (GLDM), and gray level run length matrix (GLRLM). The selected features and their corresponding nonzero weighting coefficients are shown in **Figure 3**. The radscore was calculated using the following formula:

$$\begin{array}{l} \text{Radscore}\;=\;0.05635\;+\;0.09305\times \text{T}2\;\text{firstorder}\_\text{Maximum}+0.0728\times \text{T}2\\ \text{glcm}\_\text{ClusterTendency}+0.04676\times \text{T}2\;\text{glcm}\_\text{InverseVariance}-0.10165\\ \times \text{ T}2\text{ glszm}\_\text{GrayLevelNonUniformity}-0.03472\times \text{T}2\\ \text{glszm}\_\mathrm{LargeAreaHighGrayLevelEmphasis}-0.08877\times \text{T}2\\ \text{gldm}\_\mathrm{DependenceNonUniformityNormalmalized}+0.03044\times \text{DWl}\\ \mathrm{firstorder}\_\mathrm{Kurtosis}-0.08385\times \text{DWI}\;\text{glcm}\_\mathrm{ClusterProminence}+0.05277\\ \times \text{ DWI}\text{ glcm}\_\mathrm{ClusterShade}+0.00252\times \mathrm{DWI}\;\text{glcm}\_\text{Idmn}-0.14647\times \text{DWI}\\ \text{glrlm}\_\text{ShorRunEmphasis}+0.11027\times \text{DWI}\;\text{glszm}\_\mathrm{ZoneEntropy}+0.06508\\ \times \text{ DWI}\text{ gldm}\_\text{LargeDependenceLowGrayLevelEmphasis}+0.06673\times \text{DWI}\\ \text{gldm}\_\text{SmallDependenceHighGrayLevelEmphasis}+0.08309\times \text{C}\\ \text{firsorder}\_10\text{Percentile}+0.03913\times \text{C}\\ \text{glszm}\_\mathrm{LargeAreaLowGrayLevelEmphasis}-0.06199\times \text{C}\;\text{glszm}\_\mathrm{ZonePercentage} \end{array}$$

### Radiomics nomogram development and validation

**Figure 4:**
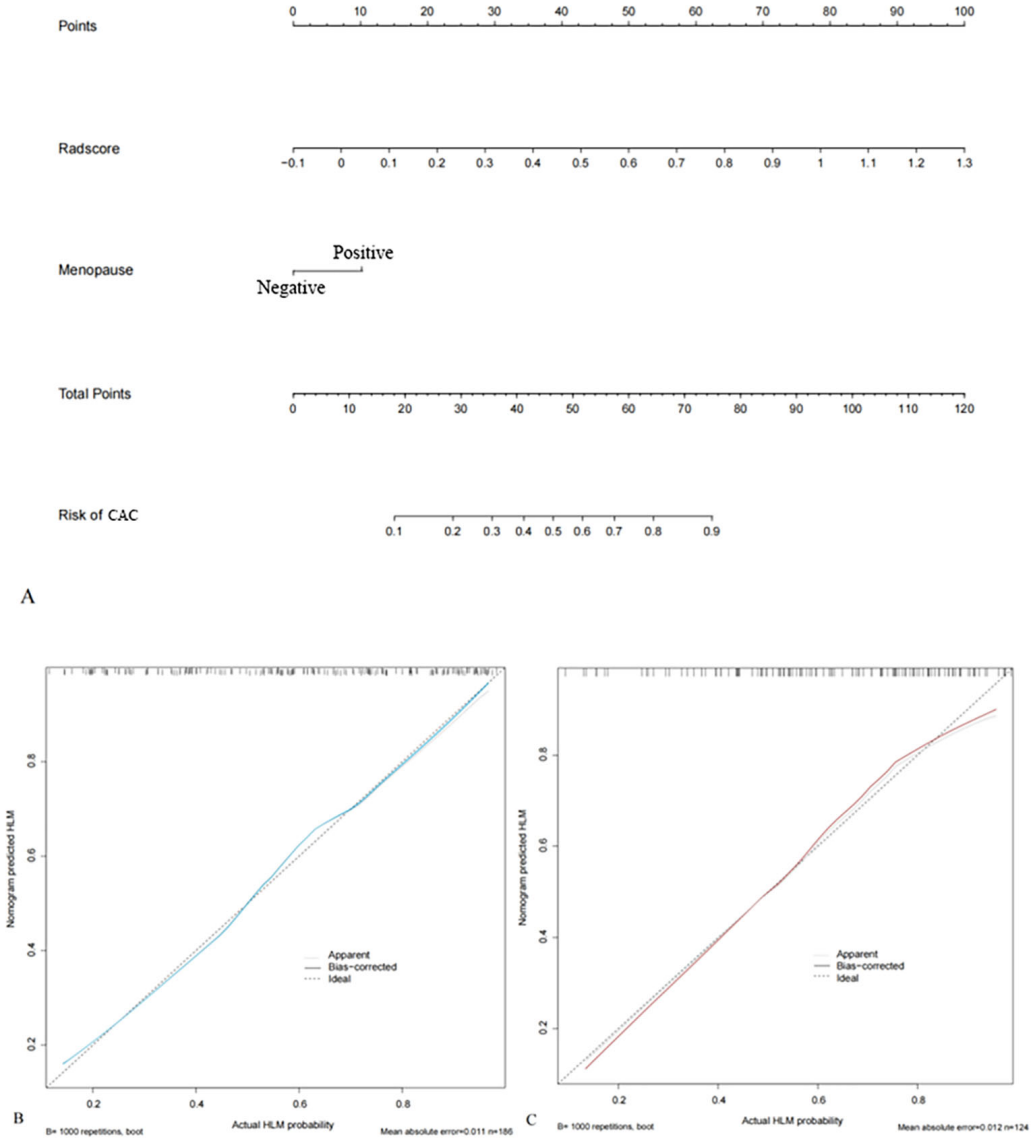
Radiomics nomograms integrating radscore and clinical independent risk factors (menopause) based on the training cohort **(A)**. The calibration curves of the training **(B)** and validation cohorts **(C)** show good agreement between the predicted and actual observations of the nomograms.

An MRI radiomics nomogram was developed by integrating the radscore with clinical independent risk factors. To minimize collinearity, tumor diameter-although a significant clinical predictor-was excluded from the final nomogram, as size-related information was already embedded in radiomic features such as firstorder_Maximum, and only menopause was retained as a clinical factor. The nomogram for predicting LVSI in early-stage CAC is shown in **Figure 4**. Calibration curves demonstrated good agreement between predicted and observed results of the nomogram in both the training and validation cohorts (**Figure 4**).

### Diagnostic performance evaluation

**Table 3** presents the diagnostic performance of the clinical model, radscore, and nomogram in the training and validation cohorts. For predicting LVSI status in early-stage CAC: The AUCs of the clinical model were 0.69 (95% CI: 0.62-0.77) in the training cohort and 0.62 (95% CI: 0.52-0.72) in the validation cohort. The AUCs of the radscore were 0.79 (95% CI: 0.73-0.86) and 0.78 (95% CI: 0.69-0.86) in the training and validation cohorts, respectively. The AUCs of the radiomics nomogram were 0.80 (95% CI: 0.74-0.86) and 0.78 (95% CI: 0.69-0.86) in the training and validation cohorts. The nomogram’s AUCs were significantly higher than those of the clinical model (both P < 0.001), although not significantly different from those of the radscore in both the training (P = 0.462) and validation cohorts (P = 0.871).

**Table 3:** Diagnostic efficacy of clinical model, radscore and nomogram in the training and validation cohorts.

Model	AUC (95% CI)	ACC	SPE	SEN	NPV	PPV	P-value^#^
Training							
Clinical risk factors	0.69 (0.62 - 0.77)	0.58	0.71	0.48	0.49	0.70	< 0.001
Radscore	0.79 (0.73 - 0.86)	0.72	0.81	0.66	0.60	0.85	0.462
Nomogram	0.80 (0.74 - 0.86)	0.74	0.79	0.70	0.65	0.83	–
Validation							
Clinical risk factors	0.62 (0.52 - 0.72)	0.53	0.43	0.59	0.40	0.63	< 0.001
Radscore	0.78 (0.69 - 0.86)	0.74	0.68	0.78	0.68	0.78	0.871
Nomogram	0.78 (0.69 - 0.86)	0.72	0.81	0.66	0.60	0.85	–

ACC, accuracy; AUC, area under the curve; 95% CI, 95%confidence interval; SPE, specificity; SEN, sensitivity; NPV, negative predictive value; PPV, positive predictive value. #, compared with nomogram by DeLong’s test; -, not applied

**Figure 5:**
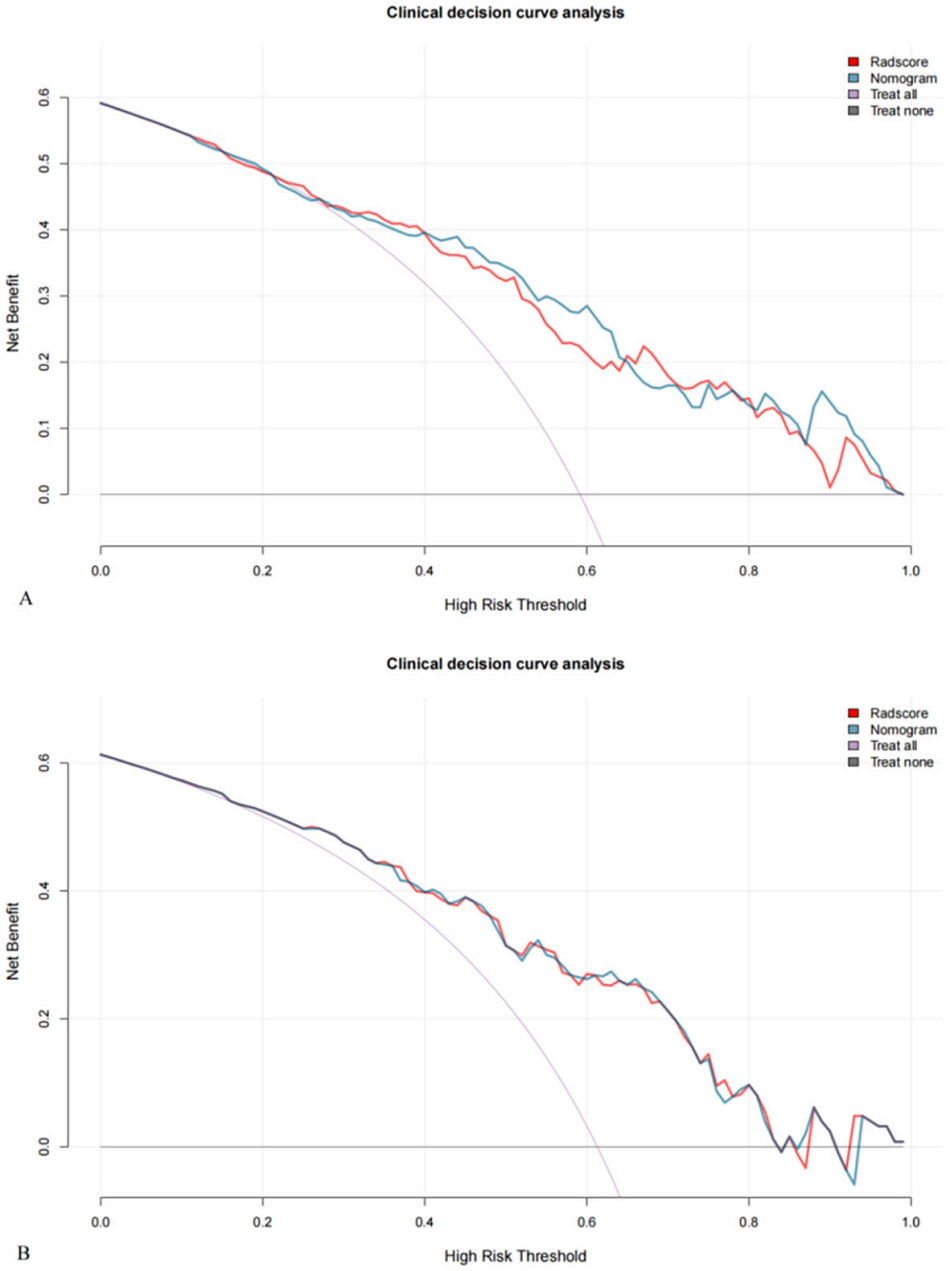
Decision curves for the training **(A)** and validation **(B)** cohorts show that nomograms achieve more clinical net benefit than full diagnosis LVSI (+) and full diagnosis LVSI (-) at a threshold probability of 1% to 86%.

Decision curve analysis (**Figure 5**) indicated that, within a threshold probability range of 1% to 86%, the radiomics nomogram and radscore provided greater clinical net benefits than the full diagnosis of LVSI (+) or LVSI (-).

## Discussion

This study developed and validated a multi-parametric MRI-based radiomics nomogram to preoperatively predict LVSI status in early-stage CAC. The results demonstrated that the nomogram, combining radiomics features and clinical factors, achieved high predictive performance. These findings highlight the potential of radiomics to enhance noninvasive risk stratification in CAC patients. Unlike prior studies that predominantly target SCC or use fewer imaging modalities, our nomogram specifically addresses CAC, a histologically distinct and more aggressive subtype. By leveraging features from T2WI, DWI, and CE-T1WI and integrating clinical risk factors, our model demonstrated superior diagnostic performance (AUC: 0.80 and 0.78) compared to prior models, which often reported AUCs in the range of 0.70-0.75. This comprehensive, CAC-specific approach fills an important gap in current radiomics research.

Previous studies commonly identify tumor size as significant predictors of LVSI in SCC. Dong S et al. indicated that LVSI was strongly associated with maximum tumor diameter in SCC (21). Chen et al.’s retrospective study showed that gross tumor volume and the maximum diameter of CC were helpful in quantitatively predicting the presence of LVS (22). The probably pathological mechanism could be that LVSI was mainly around the tumor. The larger the tumor size, the deeper the surrounding invasion and there was more likely to involve LVSI (10). Burghardt E et al. reported that tumor volume has been applied for the evaluation of patients with early invasive CAC (23). In this study, although tumor diameter was statistically significant, it was excluded from the final nomogram due to collinearity with radiomics features encoding size and intensity (e.g., firstorder_Maximum). Retaining diameter would risk inflating the model through feature redundancy. The nomogram thus reflects the added discriminative power of radiomics texture and intensity descriptors beyond simple morphometrics, particularly in tumors with heterogeneous architecture like CAC. Menopause emerged as an other independent clinical predictor of LVSI. This finding aligns with previous studies suggesting that hormonal changes may influence tumor biology, promoting aggressiveness and metastatic potential in postmenopausal women. Similar studies in SCC have also identified menopausal status as a significant factor (24).

By integrating multi-parametric MRI sequences (T2WI, DWI, and CE-T1WI), this study captured a comprehensive range of tumor characteristics. Unlike studies that often focus on fewer imaging modalities, this approach leverages the full spectrum of MRI data to enhance predictive power. The inclusion of DWI and ADC-based features aligns with previous findings that diffusion metrics are particularly effective in predicting LVSI (25). Seventeen radiomics features, including first-order and texture metrics (GLSZM, GLCM, GLDM, and GLRLM), were selected for LVSI prediction. Texture features such as GLCM_ClusterTendency and GLSZM_GrayLevelNonUniformity may reflect intratumoral heterogeneity, which correlates with glandular irregularity, necrosis, and microvascular infiltration seen in CAC with LVSI. CAC’s glandular architecture often leads to heterogeneous signal intensities on MRI, which radiomics can capture through such features. These patterns may indirectly reflect histologic indicators of aggressiveness and vascular invasion, supporting their relevance in LVSI prediction. While similar features have been reported in SCC studies, which demonstrated the association between GLSZM, GLDM and GLRLM-derived heterogeneity metrics and LVSI (26). This study is unique in demonstrating their predictive value in CAC, where glandular histology and more aggressive behavior may influence radiomics signatures. CAC’s glandular structure introduces more pronounced heterogeneity and texture variations compared to SCC. This may explain the stronger association between LVSI and texture features like GLSZM in this study. This result highlights the complexity of CAC, where LVSI prediction is complicated by unique biological pathways.

The nomogram achieved AUCs of 0.80 and 0.78 in the training and validation cohorts, respectively, significantly outperforming the clinical model alone. Although the diagnostic performance of the nomogram was only slightly improved compared to the radscore alone, the inclusion of clinical variables enhances interpretability and usability in real-world settings. The graphical format of the nomogram facilitates easier clinical decision-making and patient counseling, which can aid in individualized treatment planning. These findings underscore the potential of radiomics for noninvasive LVSI prediction, enabling early risk stratification. Li et al. (27) developed a nomogram by combining the CE-T1WI sequence and whole red blood cell count to discriminate between LVSI and non-LVSI patients, with AUCs of 0.75 and 0.73 in the training and validation cohorts, respectively. In contrast to previous radiomics investigations, this study boasted a larger sample size and a stronger focus on CAC patients, resulting in better predictive power (28). However, this study extends the application to CAC, a less common and more aggressive subtype, addressing a gap in existing literature. Compared to studies by previers studies, our model may be attributed to the exclusive focus on CAC and use of multi-parametric MRI, allowing better texture characterization of glandular tumor heterogeneity. Additionally, our sample size is among the largest in CAC-focused radiomics literature, increasing model stability.

The current study had several limitations. First, although our sample is the largest to date focused on early-stage CAC, it remains from a single institution, and the lack of external validation and the moderate size of the validation cohort (n = 124) may introduce overfitting, particularly in high-dimensional radiomics modeling. Future multi-center prospective studies are needed to confirm the reproducibility of our model across diverse imaging protocols and populations. Second, only one clinical risk factor was included, which did not enhance much of the predictive capability of the nomogram compared to the radscore alone. This observation may be attributed to the CAC subtype of patients in this study. Third, blood tumor markers, including CA125, CA199, and CEA, as well as genomic and proteomic features, were not included in this study. Fourth, all images were acquired from a single 3T scanner. Therefore, we could not assess feature robustness across different vendors or protocols. This restricts the clinical transferability of our radiomics model and warrants further validation under scanner variability. Fifth, tumors <1 cm were excluded due to segmentation limitations, potentially introducing selection bias. These smaller tumors may exhibit lower LVSI prevalence, and excluding them could skew results. Although inter- and intra-observer reproducibility was high, the manual segmentation process still carries some subjectivity. Importantly, radiologists performing segmentation were blinded to LVSI status to mitigate incorporation bias. Finally, although decision curve analysis supports the potential clinical utility of our model, direct comparison with visual MRI interpretation by expert radiologists was not performed. Such comparison is essential to establish the incremental value of radiomics in real-world decision-making and will be the focus of future prospective trials.

In conclusion, we developed a radiomics nomogram by integrating multi-parametric MRI radiomics and clinical independent risk factors, that can preoperatively and non-invasively predict LVSI status in patients with early-stage CAC, thereby help gynecologist in formulating individualized treatment strategies.

## Data Availability

The original contributions presented in the study are included in the article/**Supplementary Material**. Further inquiries can be directed to the corresponding authors.
