# Supplementary material for: Multi-parametric MRI-based radiomics nomogram for predicting lymphovascular space invasion in early-stage cervical adenocarcinoma
Source: Front Oncol. 2025 Aug 21;15:1612691. doi: 10.3389/fonc.2025.1612691 (PMC12409719; doi:10.3389/fonc.2025.1612691)
Supplement: Supplementary file 1 [file Table1.docx]

Supplementary Table 1 The diagnostic efficacy of 17 radiomics features

| Radiomics feature | Training cohort | Validation cohort |
| --- | --- | --- |
|  | AUC (95% CI) | AUC (95% CI) |
| "T2 firstorder_Maximum" | 0.60 (0.52 - 0.68) | 0.59 (0.49 - 0.69) |
| "T2 glcm_ClusterTendency" | 0.51 (0.43 - 0.60) | 0.56 (0.46 - 0.66) |
| "T2 glcm_InverseVariance" | 0.53 (0.45 - 0.62) | 0.51 (0.41 - 0.62) |
| "T2 glszm_GrayLevelNonUniformity" | 0.60 (0.52 - 0.69) | 0.58 (0.48 - 0.69) |
| "T2 glszm_LargeAreaHighGrayLevelEmphasis" | 0.61 (0.52 - 0.69) | 0.58 (0.47 - 0.68) |
| "T2 gldm_DependenceNonUniformityNormalized" | 0.58 (0.50 - 0.67) | 0.54 (0.43 - 0.63) |
| "DWI firstorder_Kurtosis" | 0.57 (0.49 - 0.66) | 0.52 (0.37 - 0.58) |
| "DWI glcm_ClusterProminence" | 0.58 (0.50 - 0.67) | 0.52 (0.40 - 0.63) |
| "DWI glcm_ClusterShade" | 0.52 (0.43 - 0.61) | 0.55 (0.44 - 0.66) |
| "DWI glcm_Idmn" | 0.58 (0.49 - 0.66) | 0.58 (0.48 - 0.69) |
| "DWI glrlm_ShortRunEmphasis" | 0.66 (0.58 - 0.74) | 0.65 (0.55 - 0.75) |
| "DWI glszm_ZoneEntropy" | 0.66 (0.58 - 0.74) | 0.50 (0.39 - 0.61) |
| "DWI gldm_LargeDependenceLowGrayLevelEmphasis" | 0.62 (0.54 - 0.70) | 0.57 (0.46 - 0.67) |
| "DWI gldm_SmallDependenceHighGrayLevelEmphasis" | 0.61 (0.52 - 0.69) | 0.56 (0.45 - 0.67) |
| "C firstorder_10Percentile" | 0.61 (0.52 - 0.69) | 0.50 (0.40 - 0.61) |
| "C glszm_LargeAreaLowGrayLevelEmphasis" | 0.54 (0.45 - 0.63) | 0.51 (0.38 - 0.59) |
| "C glszm_ZonePercentage" | 0.58 (0.49 - 0.67) | 0.50 (0.40 - 0.61) |

AUC; area under the curve; 95% CI; 95%confidence interval.

Compared with nomogram by DeLong's test; -, not applied
